# Effect of radiative and nonradiative energy transfer processes of light-emitting diodes combined with quantum dots for visible light communication

**DOI:** 10.1186/s11671-023-03812-w

**Published:** 2023-03-02

**Authors:** Wen-Hao Chiang, Yu-Hsiang Chang, Chien-Chung Lin, Hao-Chung Kuo, Gong-Ru Lin, Jian-Jang Huang

**Affiliations:** 1 Graduate Institute of Photonics and Optoelectronics, National Taiwan University, Taipei, 10639 Taiwan; 2 Department of Photonics and Graduate Institute of Electro-Optical Engineering, College of Electrical and Computer Engineer, National Yang Ming Chiao Tung University, Hsinchu, 30010 Taiwan; 3 Department of Electrical Engineering, National Taiwan University, Taipei, 10639 Taiwan

**Keywords:** LED, VLC, PhC

## Abstract

Though light-emitting diodes (LEDs) combined with various color conversion techniques have been widely explored for VLC (visible light communication), E-O (electro-optical) frequency responses of devices with quantum dots (QDs) embedded within the nanoholes have rarely been addressed. Here we propose LEDs with embedded photonic crystal (PhC) nanohole patterns and green light QDs for studying small-signal E-O frequency bandwidths and large signal on–off keying E-O responses. We observe that the E-O modulation quality of PhC LEDs with QDs is better than a conventional LED with QDs when the overall blue mixed with green light output signal is considered. However, the optical response of only QD converted green light shows a contradictory result. The slower E-O conversion response is attributed to multi-path green light generation from both radiative and nonradiative energy transfer processes for QDs coated on the PhC LEDs.

## Introduction

Visible light communication (VLC), a wireless communication technology that combines solid-state lighting and data transmission, has attracted tremendous attention recently because of its advantages, such as license-free, low-cost, and high transmission bandwidth [1–5]. Traditionally, white light is the most commonly used indoor lighting, composed of LED (light-emitting diode) generated blue light and phosphor-converted yellow light. The data transmission bandwidth using such a white light source is limited by phosphor [6, 7]. Recently, with the rapid development of various types of colloidal quantum dots (QDs), color conversion from QDs has widely been explored for solid-state lighting and micro-LED displays [8–11]. The E-O conversion bandwidth and the quality of large-signal transmission using blue LEDs combined with QDs for VLC were thus studied by many researchers [12–14]. For example, the optical bandwidth of blue LEDs with QDs was compared with blue LEDs with yellow phosphors. A bandwidth increase from 1.7 to above 2.6 MHz was achieved when the phosphor was replaced by the QDs [15]. In addition, using digital modulation techniques such as on–off keying (OOK), data rates of up to 280 Mb/s were realized from a micro-LED with perovskite QDs [16].

For most reports on the optical bandwidth of QDs, the solid-state light sources were spatially apart from the QDs [16, 17] or by QD coating on the LED surface [12]. A longer wavelength signal is generated by a radiative energy transfer process that converts short wavelength light to long wavelength. The E-O response time is determined by the carrier injection to the LED active region (carrier transit time across the semiconductor and resistance–capacitance (RC) time delay), carrier lifetime before short wavelength photons are generated, and the radiative energy transfer time when the electrons in QDs are excited and converted to long wavelength photons, etc. In addition to the radiative transfer, the energy transfer process can be nonradiative, a so-called *Förster* resonance energy transfer (FRET). When the QDs are close to the LED active region, electrons can directly transfer to QDs without going through short wavelength photon generation and electron excitation in the QDs [18]. Intuitively such a process is faster than the radiative transfer. The bandwidth, under the circumstance that all the QDs are adjacent to LED active region, thus potentially increases.

FRET has been investigated by embedding QDs in the LED nanostructures. For example, by bringing colloidal nanocrystals (NQDs) to the deep etched patterns in a LED, a twofold enhancement of the colloidal NQD emission is demonstrated and attributed to non-radiative energy transfer [19]. In addition, even though LEDs with photonic crystals (PhCs) have been explored for VLC [20–22], the study of QDs embedded within the nanopatterned LED active region for E-O modulation is rarely studied.

In this study, the small-signal E-O bandwidth and large-signal non-return-to-zero OOK (NRZ-OOK) E-O conversion of PhC LEDs embedded with QDs were investigated. Even though the E-O bandwidth and OOK modulation quality of PhC LEDs with QDs are better than those of a conventional LED with QDs when the total light output signal is considered, the optical response of only QD converted green light reveals completely different results. Green light generation from both radiative and nonradiative energy transfer for QDs coated on the PhC LEDs slows down the E-O signal conversion process. The mechanisms of the distinct behaviors of blue mixed with green and only green light are studied.

## Experimental

**Fig. 1:**
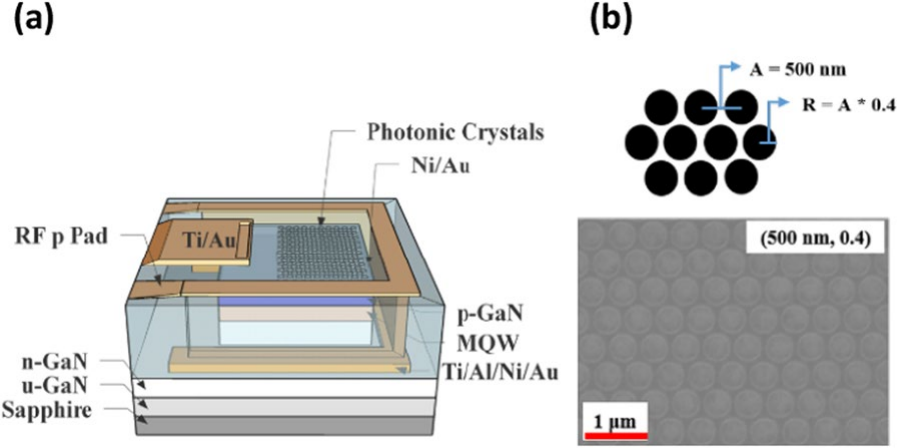
**a** Schematic structure of the PhCLED. **b** SEM image of the PhCs hexagonal nanohole arrays. The period of the nanohole, A, and the ratio of nanohole radius, R, to nanohole period are labeled

Devices studied in this work are a lateral LED structure embedded with PhC nanoholes and green colloidal QDs. The epi-structure was grown on the sapphire substrate by metal–organic chemical vapor deposition (MOCVD) and is composed of a GaN buffer, 2-μm Si-doped n-type GaN, 10 periods of InGaN/GaN multiple quantum wells (MQWs) with light emission at the wavelength of around 443 nm, and a 104 nm-thick Mg-doped p-type GaN layer. A lateral profile of the device structure is shown in Fig. 1a. The fabrication was started from first depositing Ni/Au (5 nm/5 nm) thin layers by electron-beam evaporation. The PhC nanohole patterns were defined by electron beam lithography followed by inductively coupled plasma reactive ion etching (ICP-RIE). The depth of PhC nanoholes is around 600 nm. A top view of the hexagonal PhC patterns on the LED mesa is shown in Fig. 1b. The period and radius of nanoholes in our design are 500 and 200 nm, respectively. Next, a mesa area of 120 × 120 μm^2^ was defined by ICP-RIE, followed by n-type contact metal, Ti/Au/Ni/Au (250/1250/500/1250 nm), evaporation and alloy at 900 °C for 30 s. The devices were then sealed by the SU-8 polymer. After opening up VIA holes, the interconnect probe pad, Ti/Au (50/1300 nm), was evaporated to finish the device fabrication. In this work, the LED with PhC nanoholes is called PhCLED, while the conventional LED of the same 120 × 120 μm^2^ mesa area but without nanoholes is called CLED.

**Fig. 2:**
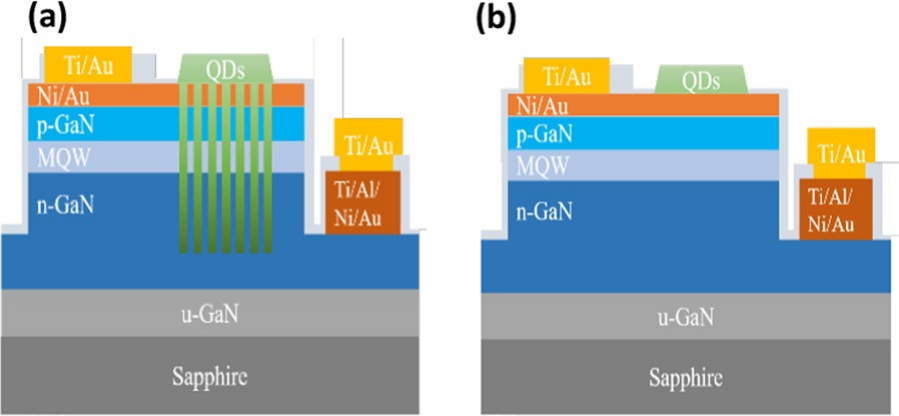
Schematic diagram of **a** QD-PhCLED, **b** QD-CLED. In this work, QD-CLEDs have three different QD coating thicknesses

Before device measurement, green colloidal CdSe QDs were pre-mixed in toluene before spraying on the light-emitting area using the aerosol jet (AJ) printer technique. Three different QD layer thicknesses were chosen for studying light transmission behaviors. Because it is not easy to quantify the exact amount of QDs on the LEDs, the effect of QDs on the optical behaviors is benchmarked by the amount of color conversion from blue to green. And the nomenclature of the conventional LED (CLED) with a QD spray time of 300, 400 and 500 ms is QD-CLED-A, QD-CLED-B and QD-CLED-C, respectively. We also prepare a PhCLED with a QD coating time of 300 ms, named QD-PhCLED in this work. The device profiles of QD-PhCLED and QD-CLED are shown in Fig. 2.

**Fig. 3:**
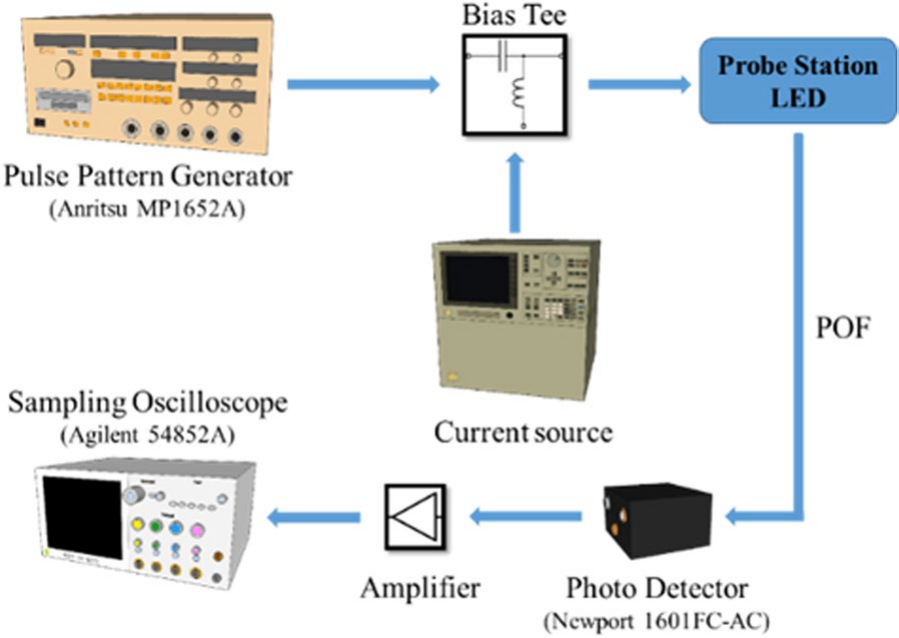
Experiment setup for E-O frequency response measurement. LED light output was collected by the plastic optical fiber (POF) and converted to the electrical signal by the photo detector

**Fig. 4:**
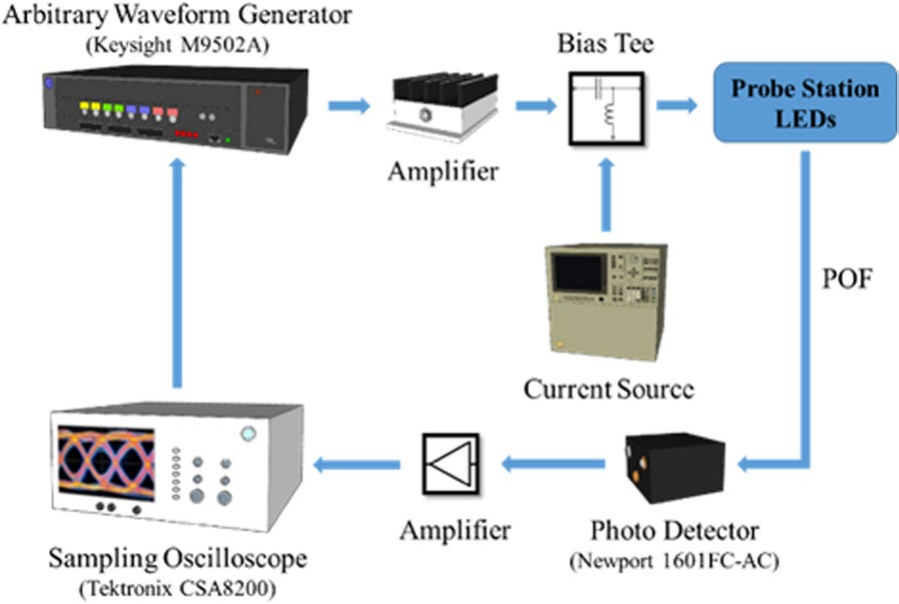
Experimental setup for OOK modulation measurement

The electrical characteristics of PhCLED and CLED were measured by the HP 4155C semiconductor parameter analyzer. As for extracting E-O (electro-optical) conversion frequency response, the measurement setup is shown in Fig. 3. A radio frequency (RF) signal generated from a pulse pattern generator was superimposed with a DC bias through a wideband bias tee. After collecting light output from the LED through an optical fiber, a high-speed silicon photodetector (New Focus, 1601FC-AC) was used to measure the optical response. Furthermore, for large-signal electrical-optical conversion, OOK modulation was performed on devices using the setup shown in Fig. 4. An arbitrary waveform generator (Keysight M9502A) delivers an electrical signal with a data rate of 100, 125, 150 and 175 Mbps. The corresponding optical response was received by the high-speed photodetector (New Focus, 1601FC-AC) with the time-domain waveforms shown on the sampling oscilloscope (Tektronix CSA8200).

## Results and discussion

**Fig. 5:**
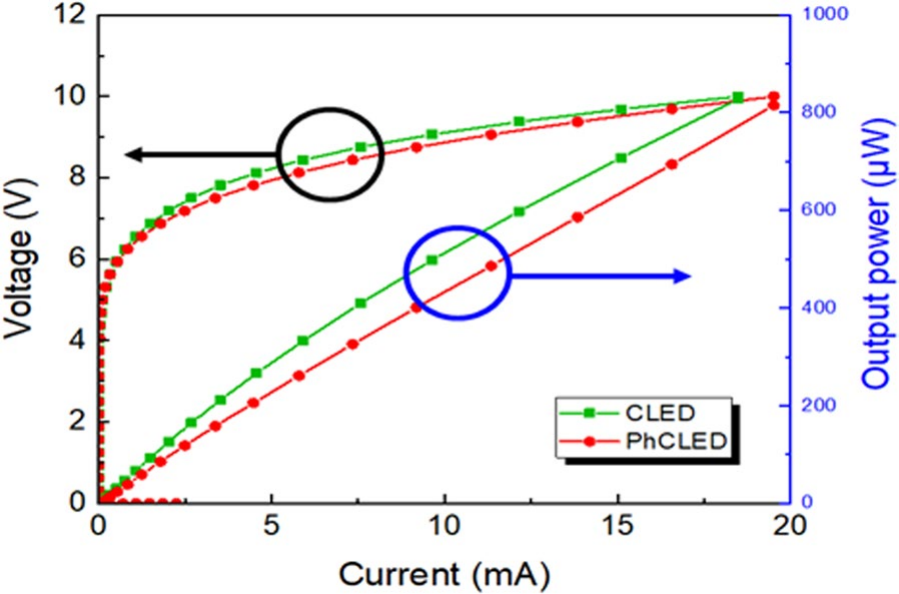
L–I–V curves of PhCLED and CLED

The luminesce–current–voltage (L–I–V) curves are shown in Fig. 5. Despite a slightly higher current level at a fixed injection current for the PhCLED, the I–V curves of the PhCLED and CLED are not much different among multiple devices tested in the samples. However, though nanoholes help to increase external light extraction [4, 20], the light output intensity of the PhCLED is lower than that of CLED because of the decreased light-emitting area with the existence of nanoholes. At an injection current of 10 mA, light output of PhCLED and CLED is 433.1 and 512.7 μW, respectively, indicating a 15.5% loss of output power for a PhCLED as compared to a CLED. In this work, we focus on data transmission and modulation in the frequency domain. The amount of light output power is not the main concern.

**Fig. 6:**
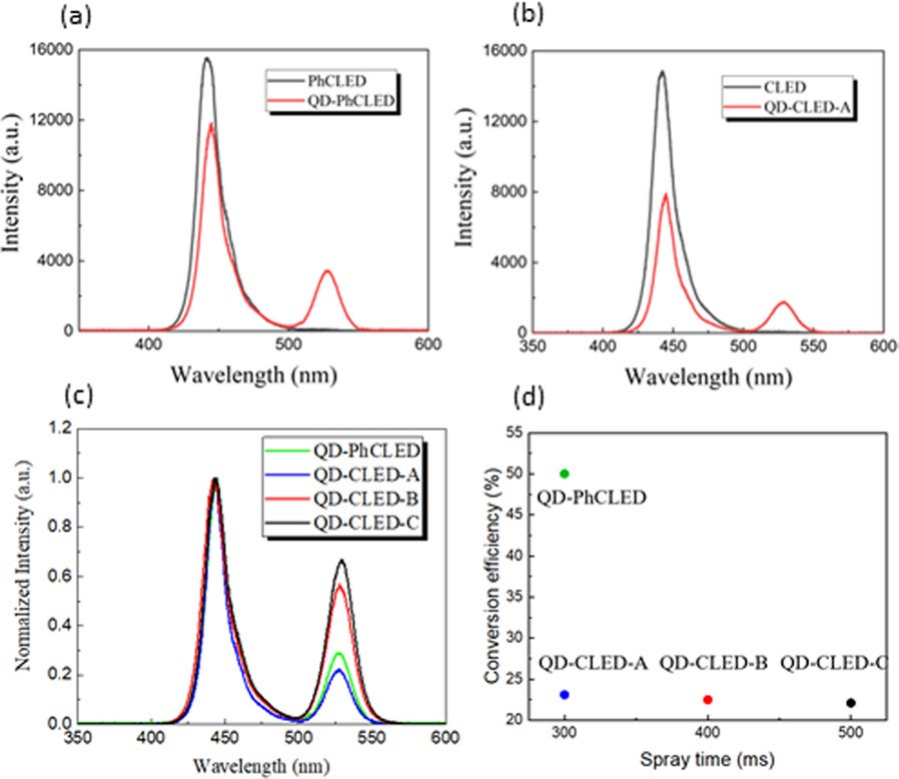
Output spectra of **a** PhCLEDs with and without QDs, **b** CLED and QD-CLED-A. **c** Normalized output spectra of the devices. **d** Conversion efficiency of the devices with QDs. Both QD-PhCLED and QD-CLED-A have the same QD spray time but the conversion efficiency of QD-PhCLED is much higher

We next characterize light output spectra of the devices using a spectrometer (Ocean Optics, HR 4000). At a bias current of 10 mA, as shown in Fig. 6, an emission peak at the wavelength of 441.3 and 442.3 nm is observed for a PhCLED and a CLED. The colloidal QD coating results in a wavelength conversion from blue to green color. For QD-PhCLED, the QD coating results in a red-shift of blue light from 441.3 to 443.7 nm and fluorescent green light emission from QDs at 527.2 nm (see Fig. 6a). While for QD-CLED, a red shift of blue light from 442.3 to 444.9 nm and green light at 527.1 nm is generated (see Fig. 6b). The blue to green intensity conversion is demonstrated from the spectra of the devices in Fig. 6c by normalizing the blue light output in the optical spectra to 1. To benchmark the dependence of color conversion on the device structure, conversion efficiencies (CEs), defined as the ratio of green light intensity increase to the blue intensity decrease at each color's peak wavelength, are plotted in Fig. 5d. The CE of the QD-PhCLED, QD-CLED-A, QD-CLED-B and QD-CLED-C is 50.0, 23.1, 22.5 and 22.1%, respectively. For a QD-CLED, the CE slightly decreases with the increase of QD spraying time because of nonradiative absorption increases with the QD layer thickness. Furthermore, a distinct difference can be observed from a much higher CE of the QD-PhCLED than QD-CLEDs. For a QD-PhCLED which QDs are embedded adjacent to the QWs (quantum wells), the green photon emission can be originated from two paths as mentioned previously: one is from the ordinary radiative energy transfer and the other is the FRET. Furthermore, the embedded and dispersed QDs in the nanoholes also significantly increase the conversion. The multi-path energy transfer from QWs to QDs thus increases CE for a QD-PhCLED.

**Fig. 7:**
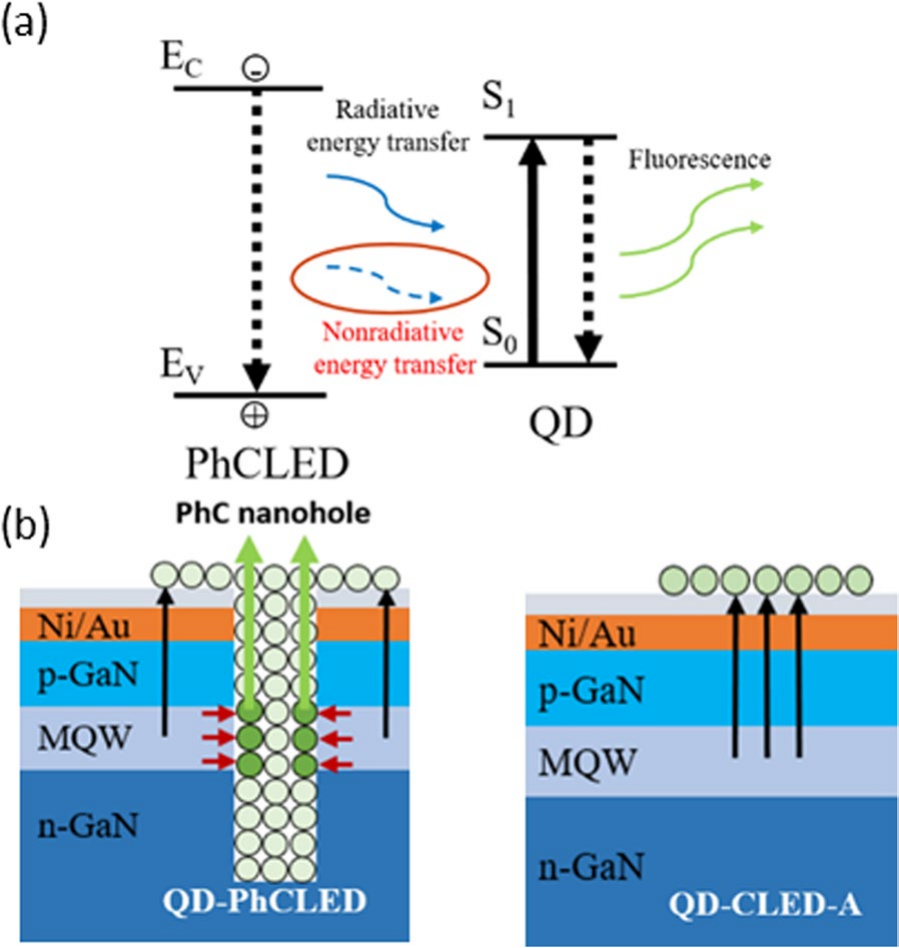
**a** Illustration of the FRET process between the MQWs in the PhCLED and QDs. **b** Energy transfer paths for the QD-PhCLED and QD-CLED. The red arrows indicate the FRET process. The green arrows are the fluorescence of QD, and the black ones are the radiative energy transfer (RET) process that converts blue light into green

The phenomenon indicated above is illustrated in Fig. 7. The FRET process between QDs (acceptors) in PhC nanoholes and QWs (donors) is indicated by the red arrows, while the blue light excited (radiative) process is shown in black arrows. The former is considered as a direct green light emission process while the later as a two-step, electron–hole generated blue and blue to green conversion, process. As a result, the conversion efficiency of QD-PhCLED is higher than that of the QD-CLED [23].

**Fig. 8:**
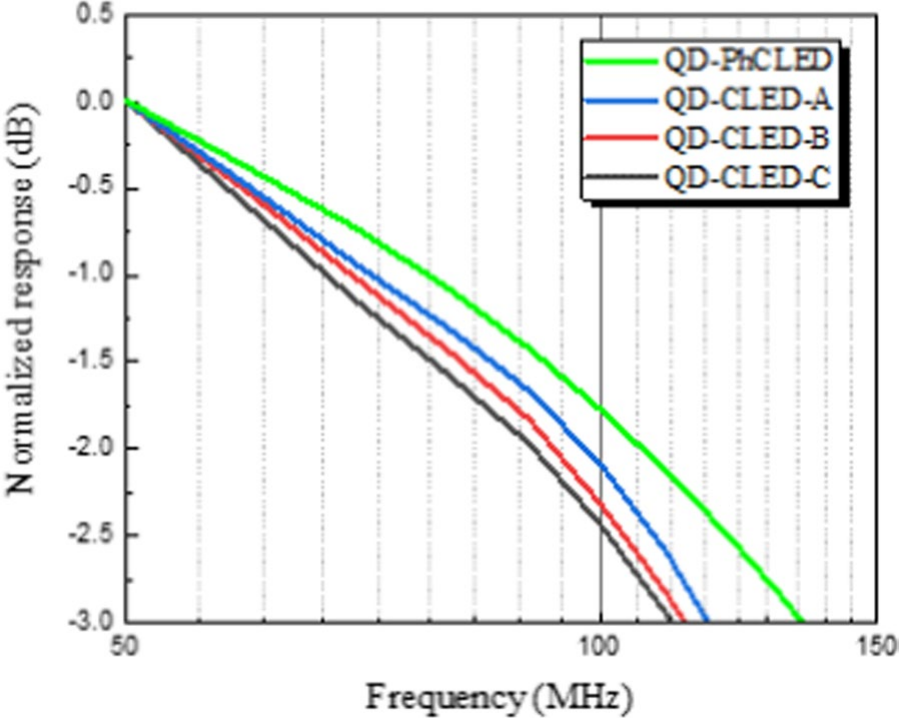
E-O frequency responses of QD-PhCLED and QD-CLEDs. Both blue and green light emissions were collected

We next conducted frequency response measurements of the devices under the injection current of 10 mA. Prior to the comparison of devices with QD coating, the − 3 dB conversion bandwidth of the PhC and CLED without QDs was extracted to be 179.5 and 150.4 MHz, respectively. The higher bandwidth of the PhCLED is mainly attributed to faster radiative carrier recombination of extracted guided modes in the photonic bands [20, 21] and a reduced effective mesa area. As for QD-PhCLED, QD-CLED-A, QD-CLED-B and QD-CLED-C, as shown in Fig. 8, the − 3 dB frequency is 136, 116, 113 and 110 MHz, respectively. Given the same mesa size of 120 × 120 μm^2^ and the same RC (resistance–capacitance) delay time for CLEDs, the decreased − 3 dB bandwidth with increasing QD layer thickness is attributed to the proportional increase of green light in the optical signal. A higher blue to green light output ratio suggests a relatively higher blue light output under the same current injection density and a higher total light output power with blue and green light in combination. Thus, among QD-CLEDs, the conversion bandwidth of QD-CLED-A is the highest while that of QD-CLED-C is the lowest. Furthermore, in Fig. 8, when the effect of PhC is considered, QD-PhCLED possesses the highest bandwidth because the blue light in the photonic bands is dominant in the E-O response.

**Fig. 9:**
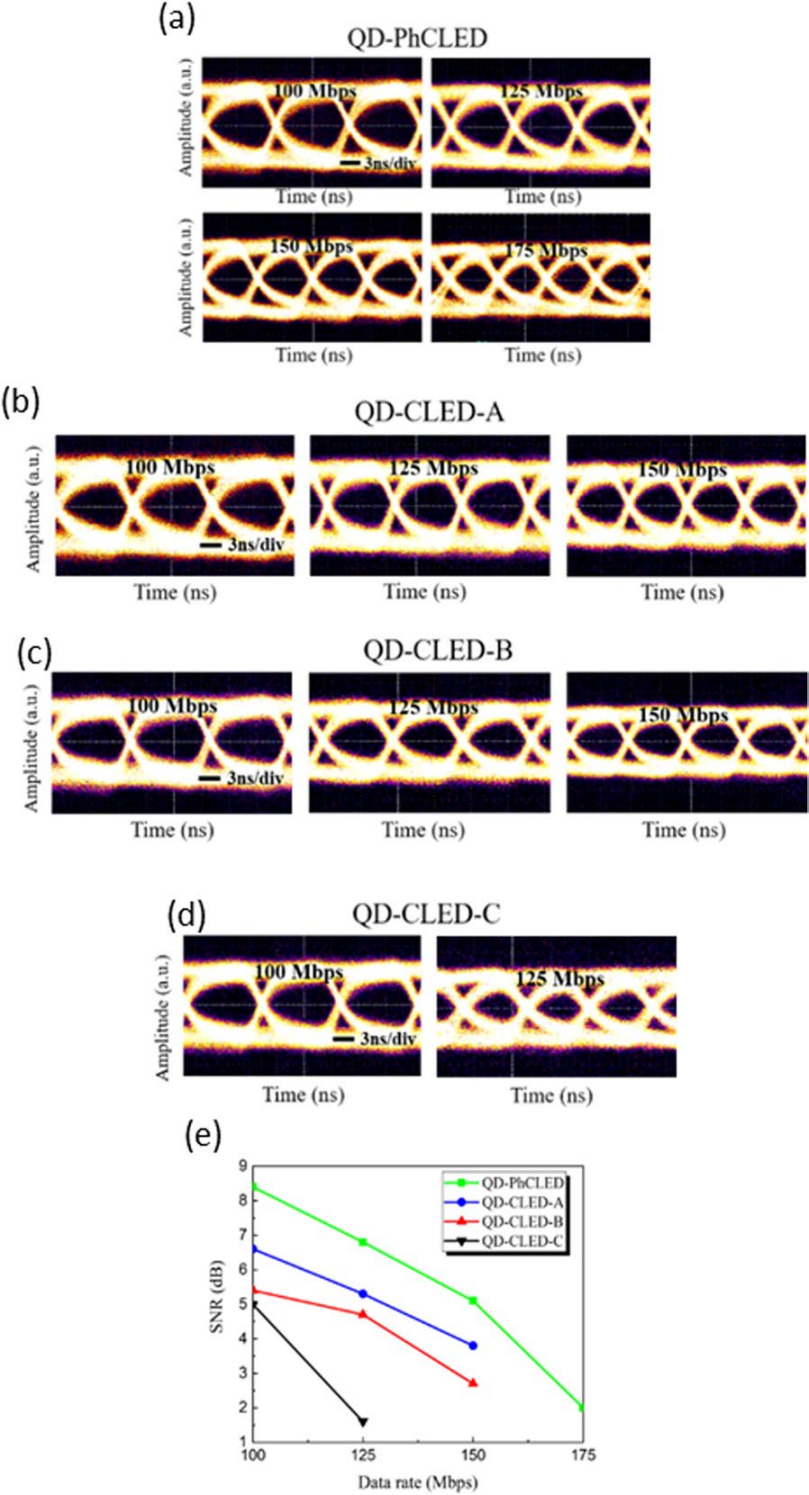
The evaluated eye diagrams of **a** QD-PhCLED, **b** QD-CLED-A,** c** QD-CLED-B and **d** QD-CLED-C. **e** The SNR performance of devices at different data rates

The NRZ-OOK technique was employed to characterize the capability of large-signal modulation. Figure 9 shows the eye diagrams of a QD-PhCLED and three types of QD-CLEDs at 100, 125, 150 and 175 Mbps. Due to the equipment limit, eye diagrams with the SNR (signal-to-noise ratio) smaller than 1 are not shown here. Even though all the devices are suitable for VLC applications at the data rate up to 100 Mbps, the performance of eye patterns degrades at a higher data rate. Figure 9e shows the corresponding SNRs of the devices at various data rates. The results are correlated to the E-O responses in Fig. 8, in which the QD-PhCLED has the best SNR performance. As for QD-CLEDs with various QD spraying times, a thin QD coating and thus a lower blue to green light conversion ratio suggests a stronger overall light intensity. We can obtain a higher SNR ratio at a giving data rate operation for QD-CLED-A. The influence of QD coating on the frequency response is very significant, for QD-CLED-C with CE slightly lower than the rest two CLEDs, the eye diagrams can only be benchmarked at data rates of 125 Mbps, beyond which at 150 and 175 MHz, the SNRs are smaller than 1.

**Fig. 10:**
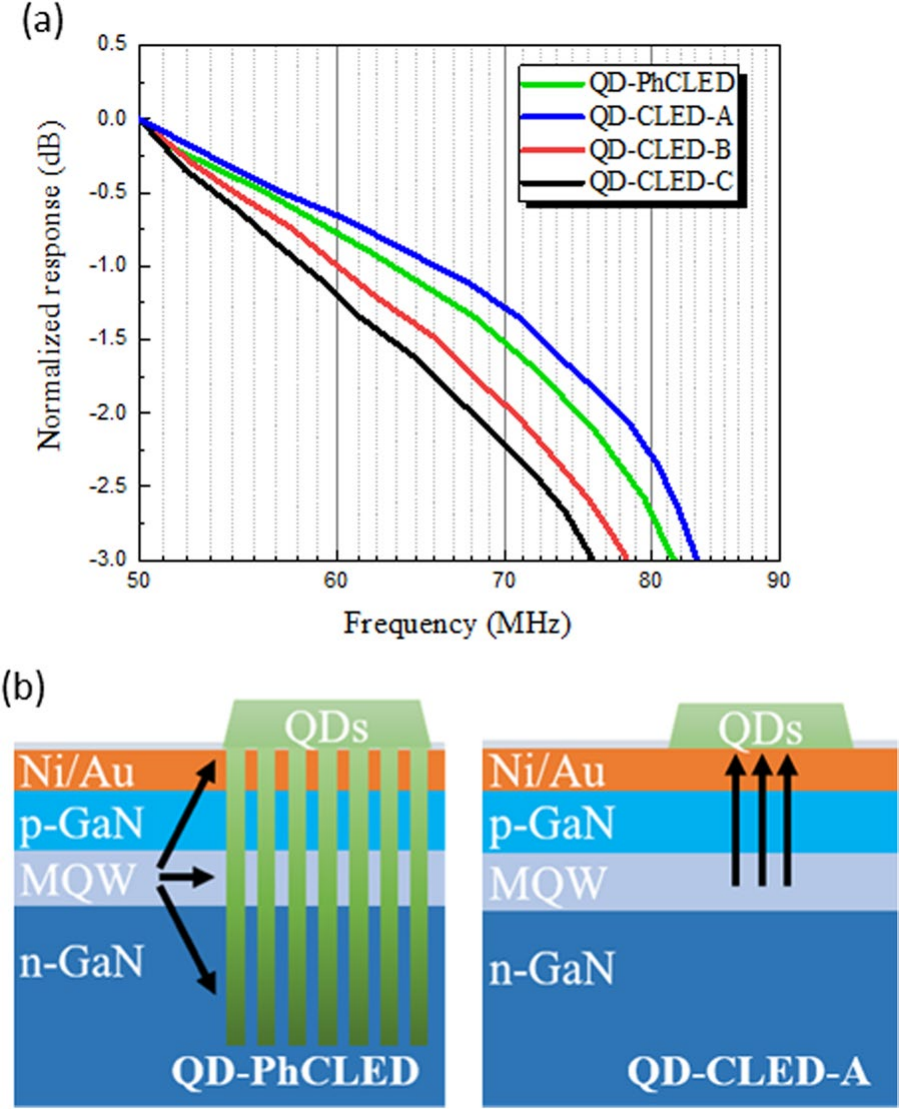
**a** Comparison of frequency responses between QD-PhCLED and QD-CLEDs. Note that in the measurement only green light was detected and analyzed. **b** Schematic illustration of energy transfer paths of QD-PhCLED and QD-CLED-A

The frequency response and OOK measurements discussed above were performed from the light sources with both blue and green light illuminated from the devices. In this session, we explore the data transmission behavior of only the QD converted green light. A green filter was placed on top of the device to remove the blue light. The E-O frequency response is shown in Fig. 10a, which suggests -3 dB bandwidth of 81, 83, 78 and 76 MHz, respectively, for QD-PhCLED, QD-CLED-A, QD-CLED-B and QD-CLED-C. Contrary to the E-O response in Fig. 8, the − 3 dB bandwidth of QD-PhCLED in Fig. 10a with only green light is slightly lower than that of QD-CLED-A. The results are mainly attributed to the FRET effect and various distances between QDs and blue light-generated MQW active region. First, in addition to transferring blue light to QDs through radiative energy, carriers can also transfer energy from QWs to QDs through nonradiative process for the QD-PhCLED. Second, for QD-PhCLED, QDs are distributed at various distances from the MQWs, as compared with nearly equal distances for QD-CLED-A (see Fig. 10b). The above two mechanisms suggest green light at different locations is excited at the different time giving an electrical signal input, thus creating phase delay of the green output signals for QD-PhCLED. A smaller -3 dB bandwidth of QD-PhCLED is observed.

**Fig. 11:**
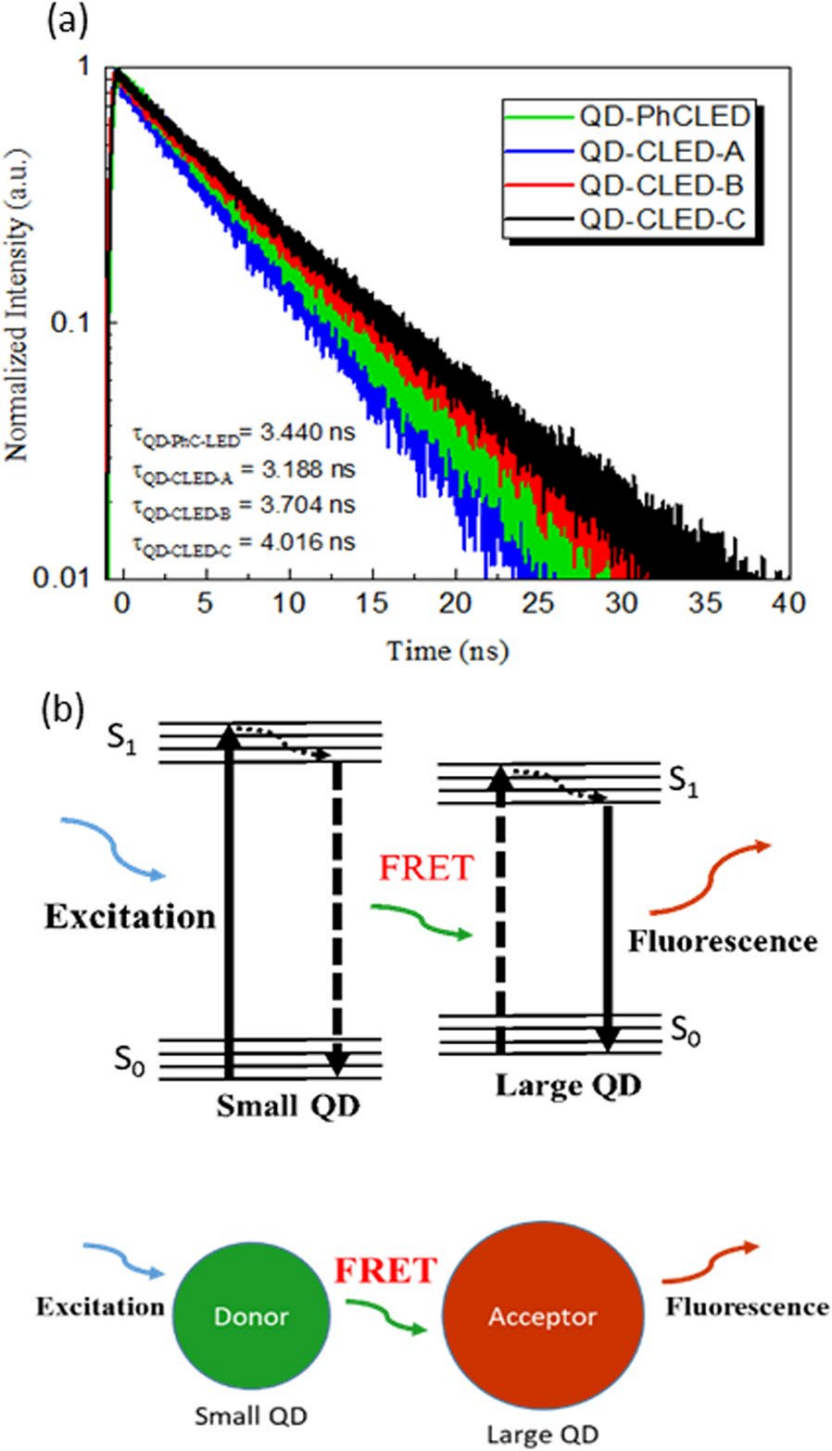
**a** TRPL decay profiles of QD-PhCLED and QD-CLEDs. **b** Schematic of re-absorption and FRET process in QDs. A small QD acts as a donor, while a large one is an acceptor. Electrons are transferred from a small QD to a large one through the FRET process

To verify the above explanation, we next carried out time-resolved photoluminescence (TRPL) measurement by filtering out the blue light. The devices were photoexcited by a 405-nm wavelength laser at an 8 MHz repetition rate. Both the blue light from the LEDs and green from QDS are collected. As shown in Fig. 11a, QD-CLED-A (blue line) possesses the shortest carrier lifetime of 3.188 ns, while QD-PhCLED (green line) has a slightly longer lifetime of 3.440 ns. The results again imply two processes are involved in the green light emission for QD-PhCLEDs. A longer carrier lifetime is thus observed [24, 25].

As for the devices with a thicker QD coating, the E-O conversion is limited by the re-absorption of the green light. The re-absorption is schematically illustrated in Fig. 11b. Since there are variations of QD diameter in the solution, shorter wavelength photons generated from a smaller QD will be re-absorbed by the QDs with a larger diameter (with a smaller bandgap), generating a longer wavelength (red-shift). The multiple conversion process increases the carrier lifetime. Another factor that influences the carrier lifetime is a thicker QD layer tends to have more QDs aggregated, increasing the chances of multiple re-absorptions.

To understand how the FRET effect and re-absorption process affect the transient E-O responses, we conducted TRPL measurement on separate samples. The lifetime of the donor without the QD donors in the nanoholes, ${\tau}_{DA}$, is 16.99 ns (donor without the acceptor), the donor with the QDs in the nanoholes (donor with the acceptor), ${\tau}_{D}$, is 14.31 ns, and the acceptor (green light from QDs) is 6.27 ns. The FRET efficiency, E, indicates the proportion of the donor carriers that have transferred excitation state energy to the acceptor and is calculated following the equation [26],

$$E = 1 - \frac{{\tau_{DA} }}{{\tau_{D} }}$$

The FRET efficiency, from the TRPL results, is estimated to be 15.77%.

## Conclusion

In conclusion, we conducted the E-O frequency response and OOK modulation measurements of the LEDs sprayed with QDs. When the overall blue and green light emission is benchmarked, devices with PhC nanoholes show a higher CE and a higher − 3 dB frequency response due to the direct carrier transport from QW to QDs, as compared with the QD-CLEDs. On the other hand, when only green light is considered by filtering out the blue color, the multi-path energy transfer to generate green color by QDs slows down the frequency response and degrades SNR in the eye diagram for the QD-PhCLED. Various energy transfer processes within QDs can be indirectly observed from TRPL and E-O frequency response. For the CLED with a thicker QD coating, the re-absorption process among QDs with various sizes and a lower CE deteriorate the frequency response. Our experimental results suggest the QD-PhCLED possesses a better bandwidth when both the blue and green light are employed for data transmission because of higher light output intensity. On the other hand, if only converted light is used for data transmission, QD-PhCLED may not be favored due to multiple radiative and nonradiative energy transfer paths, increasing the carrier lifetime.

## Data Availability

Data underlying the results presented in this paper are not publicly available at this time but may be obtained from the authors upon reasonable request.
